# X-ray induced singlet oxygen generation by nanoparticle-photosensitizer conjugates for photodynamic therapy: determination of singlet oxygen quantum yield

**DOI:** 10.1038/srep19954

**Published:** 2016-01-28

**Authors:** Sandhya Clement, Wei Deng, Elizabeth Camilleri, Brian C. Wilson, Ewa M. Goldys

**Affiliations:** 1 Australian Research Council Centre of Excellence for Nanoscale BioPhotonics, Department of Physics and Astronomy, Macquarie University, NSW 2109, Australia; 2 Department of Medical Biophysics, University of Toronto/University Health Network, Ontario, Canada

## Abstract

Singlet oxygen is a primary cytotoxic agent in photodynamic therapy. We show that CeF_3_ nanoparticles, pure as well as conjugated through electrostatic interaction with the photosensitizer verteporfin, are able to generate singlet oxygen as a result of UV light and 8 keV X-ray irradiation. The X-ray stimulated singlet oxygen quantum yield was determined to be 0.79 ± 0.05 for the conjugate with 31 verteporfin molecules per CeF_3_ nanoparticle, the highest conjugation level used. From this result we estimate the singlet oxygen dose generated from CeF_3_-verteporfin conjugates for a therapeutic dose of 60 Gy of ionizing radiation at energies of 6 MeV and 30 keV to be (1.2 ± 0.7) × 10^8^ and (2.0 ± 0.1) × 10^9^ singlet oxygen molecules per cell, respectively. These are comparable with cytotoxic doses of 5 × 10^7^–2 × 10^9^ singlet oxygen molecules per cell reported in the literature for photodynamic therapy using light activation. We confirmed that the CeF3-VP conjugates enhanced cell killing with 6 MeV radiation. This work confirms the feasibility of using X- or γ- ray activated nanoparticle-photosensitizer conjugates, either to supplement the radiation treatment of cancer, or as an independent treatment modality.

Photodynamic therapy (PDT), a clinical treatment for cancer, localized infections^1^, macular degeneration and other medical conditions, uses photosensitizer molecules (PS) and visible or near-infrared light to destroy cells by photogeneration of one or more reactive oxygen species (ROS)^2^,^3^,^4^. The photophysical mechanisms involve absorption of light by the ground-state PS molecule^5^,^6^ and subsequent energy transfer, generating ROS such as superoxide ions, hydrogen peroxide, hydroxyl radicals and singlet oxygen (^1^O_2_). The excited PS can generate cytotoxic ROS through type I and/or type II reactions. In a type I reaction, electron transfer from the excited PS to the surrounding biomolecules generates free radicals. These react with available oxygen, producing superoxide radical anions. Further addition of a proton can lead to the formation of hydrogen peroxide or biologically highly-reactive hydroxyl radicals^7^. Alternatively, in a type II reaction, the excited triplet-state of the PS can transfer energy directly to ground-state molecular oxygen, ^3^O_2_ in the cells or tissues to generate ^1^O_2_^8^,^9^. Singlet oxygen is highly reactive and causes lethal damage to cells depending on its intracellular localization, for example by damaging various cell membranes^6^,^10^.

The main factors determining the effectiveness of the photodynamic therapy include the type of photosensitizer, its concentration and cellular localization, the wavelength and irradiance of the excitation light, the concentration of available molecular oxygen, as well as the intrinsic photosensitivity of the target cells or tissues^11^,^12^,^13^. Most clinical applications use treatment light in the wavelength range of ~630–800 nm to achieve the deepest tissue penetration, and several clinical photosensitizers available have significant absorption bands in this region^14^. However, the effective depth of treatment is typically less than 1 cm^15^, so that optical fiber light delivery to deep-seated or larger tumors, or alternative nanoparticle strategies^16^,^17^ may be required^18^.

One possible approach to overcome this limitation is to use X-rays and/or γ-rays which are able to penetrate deeply into the tissue^19^. This idea has been introduced by Chen *et al*.^20^, who proposed to utilize scintillating nanoparticles to transduce ionizing radiation into visible light^18^ that, in turn, can activate adjacent PS molecules. This group subsequently reported X-ray-induced generation of ^1^O_2_ from LaF_3_:Tb^3 + ^nanoparticles conjugated to the photosensitizer meso-tetra (4-carboxyphenyl) porphine (MTCP)^21^ and from ZnO nanoparticles conjugated to meso-tera (o-amino phenyl) porphyrin (MTAP)^22^. This approach could potentially be therapeutically significant, because photodynamic activation concurrent with radiotherapy may act synergistically and yield enhanced biological responses.

In order to be able to effectively interact with a PS molecule and efficiently generate ^1^O_2_, the scintillating nanoparticles must meet several criteria. Firstly, they must strongly absorb the ionizing radiation, noting that this interaction generally decreases with increasing X-ray energy beyond the photoelectric absorption peaks (80 keV for CeF_3_^23^). Secondly, it is important that the nanoparticles absorb ionizing radiation more strongly than the surrounding tissue, so that the resulting dose partitioning reduces the radiation damage to the tissue for a given incident radiation dose. Thirdly, the nanoparticles should have high scintillation quantum yield, defined as the number of visible photons generated by absorption of a single high-energy photon. Finally, the scintillation emission spectrum and the PS absorption spectrum need to overlap significantly. We define here the X-ray singlet oxygen quantum yield, η, as the number of singlet oxygen molecules generated upon absorption of a single X- or γ-ray photon per unit photon energy. The value of η is then a key parameter governing the effectiveness of photodynamic therapy mediated by ionizing radiation. To the best of our knowledge, only one report^24^ has estimated η in a scintillating (LaF_3_) nanoparticle-PS system. The estimate was based on theoretical modelling of X-ray absorption in nanoparticles and assuming 50% conversion of X-ray energy into visible photons. With this assumption and for a radiation dose typically used in cancer radiotherapy, the estimated number of ^1^O_2_ molecules produced in irradiated cells appeared sufficient to enable successful PDT therapy. However, there have been no reports of experimentally quantifying the X-ray singlet oxygen quantum yield in scintillating nanoparticle-PS conjugates, which is the primary objective of the current work.

Determining the ^1^O_2_ quantum yield requires probing the concentration of this transient species with a short lifetime of less than 1 μs. Direct, but technically demanding approaches include EPR spectroscopy^25^,^26^ and near-infrared ^1^O_2_ → ^3^O_2_ luminescence emission at 1270 nm^6^,^27^. Recently, a variety of high-sensitivity fluorescence probes for detecting reactive oxygen species have been introduced. These enable ^1^O_2_ quantification by using standard fluorometry or fluorescence imaging^28^,^29^,^30^,^31^, despite the fact that the concentration of ^1^O_2_ concentration in biological environments is very low^32^. These probes include 1, 3-diphenylisobenzofuran (DPBF), 9-[2-(3-carboxy-9, 10-dimethyl)anthryl]-6-hydroxy-3*H*-xanthen-3-one (DMAX), 9-[2-(3-carboxy-9,10-diphenyl)anthryl]-6-hydroxy-3*H*-xanthen-3-one (DPAX) and Singlet Oxygen Green Sensor (SOSG). SOSG used in this work is a commercial probe identified^33^ to be fluorescein covalently bound with an anthracene moiety; its chemical formula has not been published. SOSG is highly specific for ^1^O_2_ compared with other ROS^34^. ^1^O_2_ reacts with SOSG to produce endoperoxides that are strongly fluorescent at 525 nm upon 488 nm excitation. In the absence of ^1^O_2,_ SOSG has a weak fluorescence that shows significant batch-to-batch variability. The fluorescence also depends on pH, both with and without ^1^O_2_. Since the pH itself may depend on nanoparticle concentration, quantitative measurements of SOSG fluorescence in the presence of nanoparticles require considerable care.

Here we demonstrate ^1^O_2_ generation from conjugates of CeF_3_ nanoparticles with verteporfin (VP), an efficient photosensitizer that works predominantly through the type II mechanism^35^ (see Fig. 1). CeF_3_ nanoparticles were been selected since CeF_3_ is an efficient scintillator^36^ that produces visible light upon X- or γ-ray excitation, with its peak emission wavelength matching well the absorption of VP. VP is a benzoporphyrin derivative that is clinically approved for PDT of neovascular macular degeneration^37^,^38^. All measurements were carried out in water as a solvent, for biocompatibility. The singlet oxygen generation from VP as well as CeF_3_-VP conjugates in water at 365 nm was first been demonstrated using SOSG, as was ^1^O_2_ generation from the conjugates under 8 keV X-ray irradiation. By using the ^1^O_2_ generated from protoporphyrin IX (PPIX), a common photosensitizer with a known UV singlet oxygen quantum yield (0.56) in water^39^,^40^, the SOSG fluorescence intensity was calibrated to yield the number of singlet oxygen molecules, enabling the X-ray singlet oxygen quantum yield of the conjugates to be calculated. Finally, we estimated the potential of singlet oxygen generation from CeF_3_-VP conjugates for effective PDT at therapeutic doses of ionizing radiation.

## Results and Discussion

**Figure 2:**
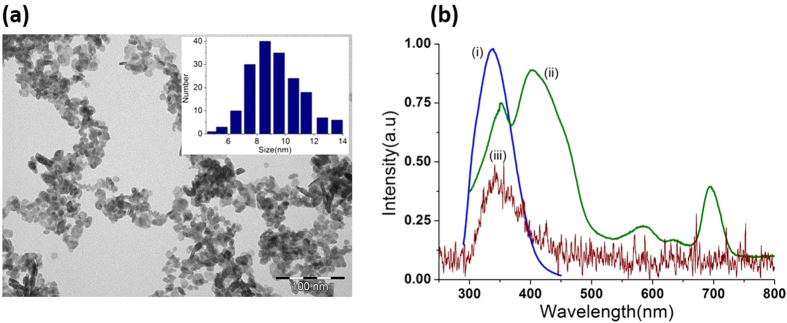
(**a**) TEM image of CeF_3_ nanoparticles(inset shows the particle size histogram) (**b**) (i) Fluorescence emission spectrum of the CeF_3_ nanoparticles under 250 nm excitation; (ii) Absorption spectrum of VP in water; (iii) Scintillation emission spectrum of CeF_3_ nanoparticles with 8 keV X-ray irradiation.

CeF_3_ nanoparticles were prepared using a co-precipitation method followed by conjugation to commercially available VP (see Materials and Methods). Figure 2(a) shows the TEM image of the nanoparticles and the size histogram. The average size of the synthesized nanoparticle is 9 ± 2 nm. As shown in Fig. 2(b), there is a high degree of spectral overlap between the CeF_3_ nanoparticle emission spectrum and the optical absorption spectrum of VP^19^, which is an important criterion for efficient photodynamic activation. The scintillation spectrum of CeF_3_ upon irradiation with 8 keV X-rays is predominantly in the UV-A range, peaking at around 340 nm, and with 30% overlap with the Soret absorption band of VP.

**Figure 3:**
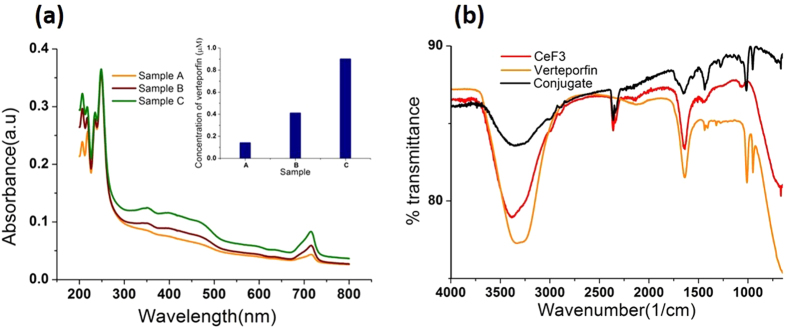
(**a**) Optical absorption spectra of CeF_3_ nanoparticles and with different concentrations of conjugated VP (0.11, 0.41, 0.9 μM in samples A, B, C, respectively, as shown in the insert). (**b**) FTIR spectra of CeF_3_, verteporfin and their conjugate (sample C).

We note that the Soret band is much stronger than the red Q-band (~690 nm) that is used for conventional visible light-mediated PDT. Varying concentrations of VP in the range 0–1 μM were conjugated with 300 μM of CeF_3_ nanoparticles, and unconjugated VP was removed by washing. These concentrations of VP ensured zero order kinetics for the concentration of SOSG used here (4 μM). The absorption spectra of the conjugates are shown in Fig. 3(a), where the peaks corresponding to VP indicate successful conjugation without any additional molecular linkage. The VP spectra in the conjugates are somewhat distorted compared to free VP, with an altered Q-to-Soret band ratio. Additional confirmation of the VP-CeF_3_ attachment was obtained by FTIR spectroscopy (See Fig. 3(b)). Although the conjugation mechanism was not conclusively determined, we note that there is electrostatic interaction between the positively-charged CeF_3_ nanoparticles and negatively-charged VP^41^. The concentration of VP in each conjugate sample was calculated from the absorption spectra (Supporting Information section S1) and it is shown in the insert to Fig. 3(a). From the VP and nanoparticle concentrations, as well as the size, density and molar mass of CeF_3_, we estimated that, on average, 31 VP molecules were conjugated to each nanoparticle in the case of Sample C. This sample had the highest concentration of conjugated VP of 0.9 μM and the highest conjugation level per single nanoparticle. The corresponding values in Samples B and C were 13 and 4 VP molecules per nanoparticle, respectively.

**Figure 4:**
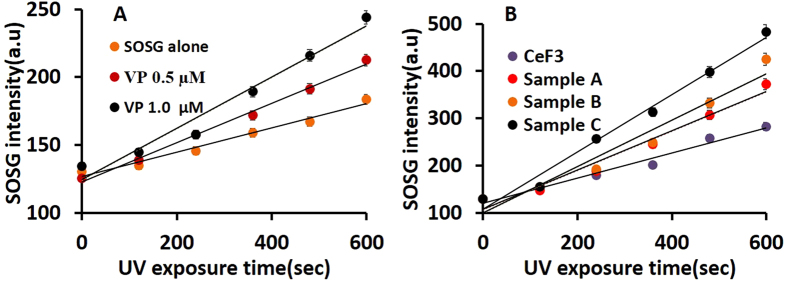
(**a**) SOSG fluorescence under UV irradiation (365 nm) for different concentrations of VP and (**b**) for different conjugates and the control samples.

The X-ray ^1^O_2_ quantum yield was then determined in several steps. Firstly, ^1^O_2_ generation from UV (365 nm) irradiation of VP and CeF_3_-VP conjugates in water was confirmed. This wavelength coincides with the VP absorption peak and also corresponds closely to the 340 nm peak emission wavelength of CeF_3_. ^1^O_2_ generation was confirmed using the SOSG probe by monitoring the enhancement of the fluorescence intensity at 488 nm excitation, integrated over the range 500–600 nm. This was done using the same concentration (4 μM) of SOSG under conditions of zero-order kinetics, while varying the concentration of the photosensitizer (see Supporting Information Section S2 and Supplementary Fig. S1). The fluorescence intensity of the SOSG emission as a function of UV irradiation time is plotted in Fig. 4(a) for VP and in Fig. 4(b) for the conjugates. In interpreting these data it is necessary to take into account the complication that SOSG itself acts as a photosensitizer under UV irradiation and that the SOSG fluorescence decreases with irradiation due to photobleaching^42^,^43^. Hence, a control sample of SOSG only was also included. The SOSG fluorescence intensities were also corrected for the inner-filter effect and for pH variations (see Supporting Information Section S3, S4, and Supplementary Fig. S2).

Figure 4(a) shows that, for a fixed concentration of VP, the SOSG intensity increases linearly with UV exposure, demonstrating that ^1^O_2_ has been generated, the amount of which also increases proportionally with VP concentration. Figure 4(b) presents the corresponding results for the conjugates, as well as for unconjugated CeF_3_ nanoparticles and the SOSG probe itself. Comparing the plots for pure SOSG (Fig. 4(a)) and pure CeF_3_ (Fig. 4(b)), we conclude that that pure CeF_3_ nanoparticles also act as a photosensitizer, which is reported here for the first time. However, CeF_3_-VP conjugates produce more ^1^O_2_ than pure CeF_3_ and higher conjugation levels lead to increased ^1^O_2_ generation, as anticipated.

**Figure 5:**
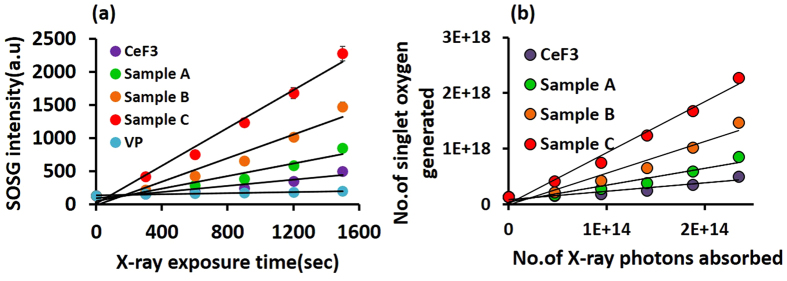
(**a**) SOSG fluorescence under X-ray irradiation for the conjugates (A–C) and control samples (pure CeF_3_ nanoparticles and pure VP). (**b**) Number of ^1^O_2_ molecules generated as a function of number of X-ray photons absorbed by the conjugated nanoparticles.

In order to quantify the generation of ^1^O_2_ under X-ray exposure and to determine the value of η, new conjugate samples containing the same amount of CeF_3_ and VP as previously were mixed with 4 μM of SOSG and exposed to X-ray irradiation. Figure 5(a) demonstrates that ^1^O_2_ is indeed generated during X-ray exposure, with a significant increase in the SOSG fluorescence compared to the nanoparticle-only and PS-only controls. In order to determine the X-ray singlet oxygen quantum yield we designed a new procedure, since the reference method^44^ cannot be used to determine this due to the absence of applicable standards. Firstly, a 1 μM concentration of the well-established photosensitizer protoporphyrin IX (PpIX) was combined with 4 μM SOSG and the ^1^O_2_ produced under UV irradiation was measured using SOSG (Supplementary Fig. S3 (a)). The total number of UV photons absorbed by the PpIX was determined by standard methods (Supporting Information Section S5 and Supplementary Fig. S4 (b)). The SOSG fluorescence intensity was then related to the number of detected singlet oxygen molecules, based on the known ^1^O_2_ quantum yield of PpIX in water (0.56) under UV irradiation (Supplementary Fig. S3(d)). Finally, the total number of X-ray photons absorbed by the conjugate was determined (See Supporting Information Section S6 and Supplementary Fig. S4).

By combining the experimental results for UV-irradiated PpIX and X-ray-irradiated conjugates (see Supporting Information section S5 and S6 for the calculation), the number of ^1^O_2_ molecules generated by X-rays has been plotted in Fig. 5(b) as a function of number of X-ray photons absorbed by the nanoparticles. From the slope of the best fit, the number of ^1^O_2_ molecules generated by each absorbed 8 keV X-ray was calculated as listed in Table 1 for each conjugate sample, together with the respective values for the X-ray ^1^O_2_ quantum yield, η. From Table 1 we conclude that, in order to produce one singlet oxygen molecule in sample C with the highest VP conjugation level achieved here, required 1.27 ± 0.08 eV of absorbed X-ray energy. For comparison, 0.98 eV is required to excite ground-state ^3^O_2_ to the singlet state ^1^O_2_^45^, which means that, in our case around 30% of the X-ray photon energy is lost through other radiative and non-radiative processes.

**Figure 6:**
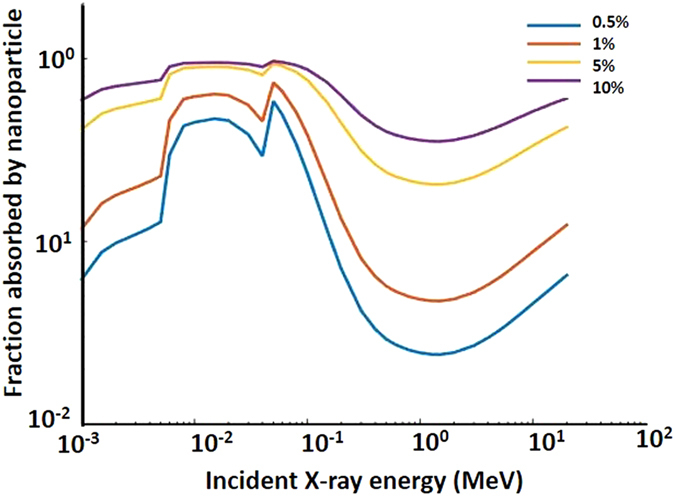
Fraction of radiation absorbed by CeF_3_ in lung tissue as a function of X-ray energy for different nanoparticle volume fractions, as shown in the insert.

The values of the X-ray induced singlet oxygen quantum yield, η, can be used to estimate the ^1^O_2_ dose achievable with CeF_3_-VP conjugates during standard cancer radiotherapy. Several effects must be taken into account in this calculation. Firstly, the nanoparticles contain heavier elements than in tissue and interact more strongly with ionizing radiation, so that they receive a higher radiation dose than the tissue for the same total incident X-ray dose. To quantify this effect we determined the partitioning of the radiation dose between the nanoparticles in the tissue and the tissue itself (see Supporting Information Section S7). The fraction of radiation energy absorbed by the nanoparticles, 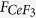 is given by:


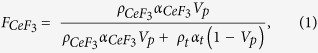


where 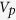 is the volume fraction of nanoparticles in the tissue (proportional to nanoparticle concentration), 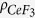
and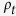 are the density of CeF_3_ and the tissue, respectively, and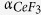 and 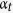 are the mass absorption coefficients of CeF_3_ and the tissue, respectively. The mass absorption coefficient of CeF_3_ for different X and γ-rays energies was obtained from the NIST database^23^ for elemental Ce and F and for an example (lung) tissue. Figure 6 shows the values of 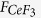 as a function of energy for different volume fractions, 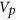; these are in agreement with earlier reports^24^.

We then assumed a nanoparticle loading of 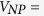
5% cell volume, as in ref. 24, noting that the relevant literature values vary from 0.1 to 33.7%. The photon energies used were based on current radiotherapy treatments; these were 6 MeV for high energy external-beam and 30 keV as representative of brachytherapy. As seen in Fig. 6, at 6 MeV the CeF_3_ nanoparticles absorb 28% of the total absorbed energy and 72% is absorbed by the tissue, whereas at 30 keV 87% of energy is absorbed by the nanoparticles versus only 13% absorbed by the tissue.

Once the dose partition is known, the energy delivered to the nanoparticles can be determined based on the radiation dose delivered to the tissue. Assuming a therapeutic tissue dose over the course of fractionated treatment of 60 Gy, the radiation energy delivered per cell was calculated as follows. The cell is assumed to be a sphere of (10 μm)^3^ with water as main constituent and with a mass of 10^−12^ kg. 60 Gy delivered dose means that an energy 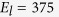
MeV is absorbed per cell. The energy per cell absorbed by the nanoparticles can be then found by the relation:


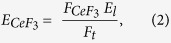


where 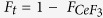. Using equation (2), the dose partition values in Fig. 6 and a 5% volume ratio yields 146 and 2510 MeV at photon energies of 6 MeV and 30 keV, respectively. It is further assumed that the number of ^1^O_2_ molecules, 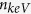, generated per keV of incident photon energy as derived above for 8 keV is the same at 30 keV, 6 MeV and all intermediate energies. This assumption is supported by the observation that the scintillation quantum yield across a wide range of materials is roughly constant (within ±20–40%) across this energy range^46^. Thus, using the previously obtained value of the X-ray induced singlet oxygen quantum yield and the value of energy absorbed by the CeF_3_ per cell during radiotherapy treatment, the number of ^1^O_2_ molecules generated per cell 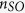 is given by:


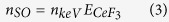


The calculated value of 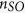 for the most efficient conjugate measured (Sample C) is then (1.2 ± 0.7) × 10^8^ for 6 MeV and (2.0 ± 0.1) × 10^9^ for 30 keV. It is of interest to compare these values to literature estimates of the singlet oxygen dose required for cell killing. The most direct measurement of photodynamic cell killing dose^47^, carried out *in vitro* in leukaemia cells PpIX as the photosensitizer (following incubation with the prodrug aminolevulinic acid) and using direct near-infrared luminescence dosimetry, showed that ~5 × 10^7 1^O_2_ molecules per cell result in 1/e clonogenic surviving fraction. Thus, 1.2 × 10^8^ to 2.0 × 10^9 1^O_2_ molecules per cell would correspond to ~10% and negligible surviving fraction, respectively. Other studies have estimated the concentration of ^1^O_2_ sufficient to cause tissue necrosis (in rat liver) to be 0.9 mM (~5 × 10^8^ molecules per cell)^48^, while the threshold dose of singlet oxygen estimated for tumour spheroids was 0.323 mM (~2 × 10^8^ molecules per cell) assuming no photosensitizer photobleaching^49^. These values are comparable to those obtained here for X-ray irradiation of the most efficient conjugates described here.

**Figure 7:**
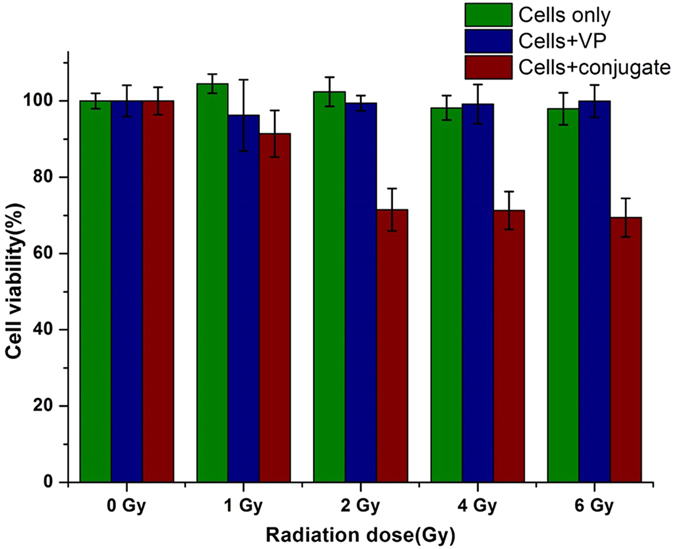
The viability of Panc 1, pancreatic cancer cells treated with CeF_3_-VP conjugate and controls (cells only, cells + VP) at different radiation doses.

We validated our approach by a radiation–induced PDT experiment at 6 MeV conducted in cell cultures, where cells were treated with CeF3-VP conjugates prior to radiation treatment. Here, we used pancreatic cancer (Panc1) and HEK293 (control) cell lines. The viability of both types of cells with a different radiation (dose up to 6 Gy) and with different dilutions of the most efficient conjugate C was determined (Supporting Information Section S8 and Supplementary Figs S5 and S6). On this basis, the optimum concentration of conjugate C (80 μM), for which both cancer and control cells have shown 100% viability, has been selected for radiation-induced PDT demonstration. The Panc 1 cells were treated with the conjugate C at 80 μM. The treated Panc1 cells and controls (Panc1 with VP only) were incubated overnight and then exposed to radiation. Figure 7 shows the viability of cells which were treated with the conjugate and their controls for different radiation dose. The viability of cells treated with the CeF_3_-VP conjugate clearly decreases at different radiation doses. For example, at 6 Gy radiation dose 32% cells were killed, which is an indication of efficient PDT with γ-radiation.

## Conclusions

Singlet oxygen generation from VP and from CeF_3_-VP conjugates was quantified using a fluorescent probe, SOSG, which is ^1^O_2_ specific, so that there was unequivocal generation of singlet oxygen upon X-ray exposure. The X-ray induced ^1^O_2_ quantum yield for the most efficient conjugate with 31 VP molecules per nanoparticle was 0.79 ± 0.05. With that information we estimate the concentration of ^1^O_2_ generated in nanoparticle-loaded tissue upon exposure to high energy (6 MeV) or low energy (30 keV) ionizing radiation. A radiotherapeutic dose of 60 Gy delivered to tissue containing a 5% volume fraction of PS-conjugated nanoparticles produced 1.2 × 10^8^ to 2.0 × 10^9 1^O_2_ molecules per cell. These values are within the range of significant cytotoxicity reported both in *in vitro* and *in vivo* for light-activated photodynamic therapy. Hence, it is conceivable that these nanoparticle conjugates could enhance the therapeutic efficacy of high-energy external-beam radiotherapy or low-energy brachytherapy through the complementary mechanism(s) of cell death between ionizing radiation (DNA damage) and photodynamic (membrane damage) treatments. This could then be exploited either to increase the anti-tumour effect or to reduce the normal tissue toxicity, especially if the conjugates have intrinsic preferential localization in tumour or are biomarker targeted. The alternative perspective is to develop X-ray activated PDT for treatment of larger inaccessible tumours that are not amenable to conventional light-activated PDT. Radiation induced PDT and cell killing has been demonstrated in a cell culture at 6 MeV radiation energy.

## Materials and Methods

VP, protoporphyrin IX and DMSO were purchased from Sigma Aldrich (Australia) and used without further purification. SOSG was purchased from Invitrogen (USA). Stock solutions of VP (3 mM) and protoporphyrin IX (3.5 mM) were prepared by dissolving 2 mg photosensitizer molecules in 1 ml dimethylsulfoxide (DMSO) and then were kept in the dark below 4 °C. The stock solution of SOSG (500 μM) was prepared by dissolving 100 μg (1 vial) in 330 μl methanol and then kept in frozen in the dark. CeF_3_ nanoparticles were prepared using a simple co-precipitation method^50^. Briefly, 6 mmol of NH_4_F was dissolved in 20 ml of methanol and the solution was heated to 70 °C. 2 ml of methanol containing 2 mmol of CeCl_3_.7H_2_O was added drop wise to the above and the mixture was stirred at 600 rpm. After 5 hrs, the CeF_3_ nanoparticles were cooled down and washed several times. Their average size was ~10 nm. A stock 5 mM suspension of nanoparticles was prepared by adding 1 mg of nanoparticles to1 ml of water.

To conjugate the nanoparticles with the photosensitizer, 500 μM of CeF_3_ and 0.5 μM of VP were mixed in a rotator at room temperature for 6 h at 200 rpm. After 18 h the mixture was centrifuged at 15,000 rpm for 20 min. The supernatant was removed and washed twice. The same procedure was repeated to conjugate the same amount of CeF_3_ with different concentrations of VP (1 and 1.5 μM). All measurements were done under oxygenated conditions.

For singlet oxygen generation measurements, 2 ml conjugate and control samples (VP only, water and CeF_3_ only) were placed in a quartz cuvette and 4 μM of SOSG was added. The samples were excited at 488 nm and the emission of SOSG in the 500–600 nm range was measured before after the UV irradiation/X-ray radiation and it was plotted as a function of time.T he increase in the emission intensity is an indication of singlet oxygen generation.

In this study we used HEK293 (ATCC CRL-1573), embryonic kidney cells as controls (normal cells) and Panc1 (ATCC CRL- 1469), epithelioid carcinoma/pancreas ductal cells as cancer cells.

Cells were subcultured and maintained in complete culture medium (Dulbecco’s modified Eagle’s medium (DMEM; Gibco, Grand Island, NY, Catalog No: 11995-065) containing 10% fetal calf serum (FCS; Gibco, Catalog No: 16000-044), penicillin/streptomycin (P/S; 100 U/ml; Gibco, Catalog No: 15240-062,). Cells were incubated at 37 °C 5% CO_2_ incubator.Passaging of cells was performed once the confluency reached 80%, cells were washed with PBS and trypsinised with Tryp LE (GIBCO, Australia, Catalog No: 12563-029). Following incubation for 5 min at 37 °C, complete medium were added to a trypsinised cells. Cell suspension was centrifuged at 500 g for 5 minutes. After removing the supernatant, cells pellet was resuspended in complete medium. The cell viability has been checked by calorimetric method using CellTiter 96® AQ_ueous_ One Solution Cell Proliferation Assay (MTS)(Promega Co;,USA,Catalog No.G3582).

The cells, normal and cancer, as well as appropriate controls (approximately 3 × 10^5 ^cells/ml) were seeded in the wells of a 96 well plate (100 μl in each) and incubated over night. These wells were exposed to different doses of radiation (1 Gy, 2 Gy, 4 Gy and 6 Gy) radiation and incubated again for 24 hrs. The MTS assay testing cell viability was carried out according to the manufacturer protocol and the absorbance at 492 nm was measured after 2 hours using plate reader. Cell viability was then calculated as a percentage of the absorbance of the untreated control, which was set to 100%.

To check the cell viability in the presence of the conjugate, Conjuate C (CeF_3_-320 μM) and its 2, 4 and 8 times dilution were added to a reduced serum medium. The normal (HEK293) and cancer cells (Panc1) were seeded in the wells as indicated earlier and incubated overnight. Then the medium was removed and added the conjugate with different dilution in a reduced serum medium and incubated overnight to ensure cell uptake. After 24 hours, the medium was removed, fresh medium was added and the MTS test was carried out as indicated earlier.

To perform PDT in cells, the optimised conjugate C at 80 μM (the maximum concentration f the conjugate which showed negligible toxicity to both normal and cancer cells) and a control amount of VP were prepared in a reduced serum medium. The Panc 1 cells were seeded in the wells of the 96 well plate (5 different plates for different radiation doses) and incubated for 24 hours. Then the conjugate with different controls were added and incubated overnight. After 24 hours, the medium was changed and the wells were exposed to different radiation doses(1 Gy, 2 Gy, 4 Gy and 6 Gy). Afterwards, the cells were again incubated overnight and an MTS assay was carried out to check the viability.

TEM image of nanoparticles were taken with PHILIPS CM10 system with an accelerating voltage of 100 kV.Fluorescence measurements were carried out using a Cary Eclipse fluorescence spectrophotometer with 5 nm spectral resolution for both excitation and collection. The absorption spectra were measured using a UV/VIS/NIR Cary dual-beam spectrophotometer with paired 1 cm path length quartz cuvettes cleaned with ethanol. UV irradiation was performed with a 365 nm high power LED with 2.4 mWcm^−2^ incident power density. X-irradiation was performed using an XPert Pro system (PANalytical, Netherlands) operating at 45 kV/40 mA. The system produced Cu-K_α_ radiation with a Ni filter to produce 8 keV X-rays. For the PDT experiment in cells with γ-ray radiation at 6 MeV, a linear accelerator (LINAC, Elekta AB, Sweden) was used to irradiate the samples. Each well in 96 well plates were CT-scanned and a radiation dose distribution was planned on an Elekta XiO planning system (Elekta AB, Sweden) to deliver a different dosage (1 Gy, 2 Gy, 4 Gy and 6 Gy) to each plate. Irradiation of the samples was carried out using 6 MV photons from anterior and posterior directed radiation fields. The absorbance in the 96 well are measured using a Fluorostar Galaxy plate reader by setting the wavelength at 492 nm with proper gain adjustment.

## Additional Information

**How to cite this article**: Clement, S. *et al*. X-ray induced singlet oxygen generation by nanoparticle-photosensitizer conjugates for photodynamic therapy: determination of singlet oxygen quantum yield. *Sci. Rep.* **6**, 19954; doi: 10.1038/srep19954 (2016).

## Supplementary Material

Supplementary Information

## Figures and Tables

**Figure 1:**
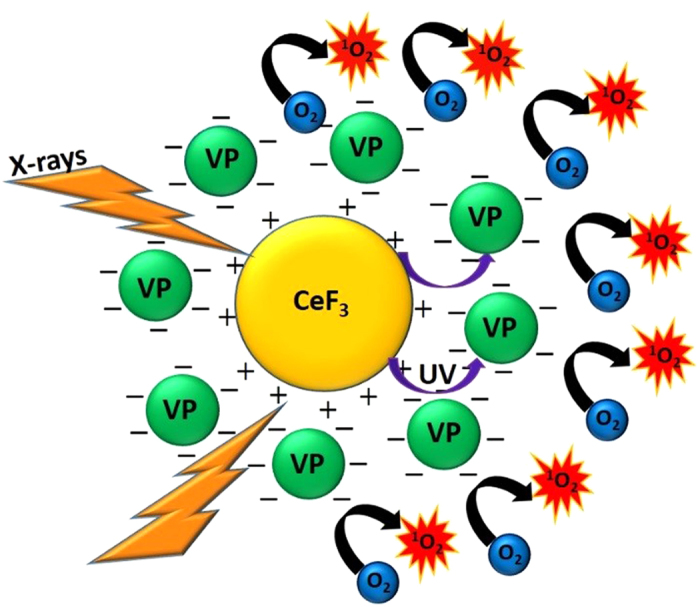
Schematic representation of singlet oxygen generation from CeF_3_-VP conjugate used in this work with X-ray radiation. Upon X-ray radiation, CeF_3_ emits UV light, which in turn, excites VP and leads to ^1^O_2_ generation.

**Table 1:** Calculated ^1^O_2_ generation from the CF_3_-VP conjugates under X-ray exposure and corresponding quantum yields. The errors originate from linear fit of data.

	**CeF3**	**Conjugate sample A**	**Conjugate sample B**	**Conjugate sample C**
^1^O_2_ molecules per absorbed 8 keV X-ray	1000 ± 170	2100 ± 280	3900 ± 470	6300 ± 380
X-ray singlet oxygen quantum yield (η)	0.13 ± 0.02	0.26 ± 0.04	0.49 ± 0.06	0.79 ± 0.05
