# Supplementary material for: X-ray induced singlet oxygen generation by nanoparticle-photosensitizer conjugates for photodynamic therapy: determination of singlet oxygen quantum yield
Source: Sci Rep. 2016 Jan 28;6:19954. doi: 10.1038/srep19954 (PMC4730142; doi:10.1038/srep19954)
Supplement: Supplementary Information [file srep19954-s1.pdf]

X-ray induced singlet oxygen generation by nanoparticle-photosensitizer conjugates for photodynamic therapy: determination of singlet oxygen quantum yield

Sandhya Clement[1] , Wei Deng[1], Elizabeth Camilleri[1], Brian C.Wilson[2], Ewa M.Goldys[1,]*

[1]Australian Research Council Centre of Excellence for Nanoscale BioPhotonics , Department of Physics and Astronomy ,Macquarie University, NSW 2109, Australia

Email: ewa.goldys@mq.edu.au

[2]Department of Medical Biophysics, University of Toronto/University Health Network, Canada

Supporting information

S1. Determination of concentration of CeF_3 and VP in the conjugate

The absorption spectra of pure CeF_3 and pure VP are first measured individually, at known concentrations, to provide reference values. The absorption spectra of the conjugates are then measured as shown in Figure 1B to find the wavelengths of the main absorption peaks for CeF_3 (λ_1= 248 nm) and for VP (λ_2 = 351 nm). The molar absorptivities of CeF_3 (ξ_c) and VP (ξ_v) are then calculated from the relevant absorption values at λ_1 and λ_2. We also record the absorbances (A) of the conjugate at these wavelengths. With these data, the concentrations of

the CeF_3 and VP in the conjugate (C_c and C_v) are calculated by solving the following system of equations

$$A^1 = \xi_c^1\, bC_c + \xi_v^1\, bC_v \tag{S1}$$

$$A^2 = \xi_c^2\, bC_c + \xi_v^2\, bC_v \tag{S2}$$

where A^1 and A^2 are absorbance of the conjugate at λ_1 and λ_2, respectively. C_c and C_v are the concentrations of CeF_3 and VP in the respective pure samples (in moles per litre), b is the path length (in cm), ξ_c^1 , ξ_c^2 are the absorptivities of CeF_3 at λ_1 and λ_2 , and ξ_v^1, ξ_v^2 are the absorptivities of VP at λ_1 and λ_2 respectively.

S2. Optimization of SOSG concentration.

We optimized the relative concentration of SOSG and VP to make sure that zero-order reaction conditions with respect to SOSG have been achieved. This means that SOSG must be in sufficient excess so that it is not markedly drawn down during singlet oxygen generation [1]. To verify that we measured the fluorescence spectra of SOSG following 365 nm photoirradiation of 1μM of VP expected to produce singlet oxygen. This was carried out for different concentrations of SOSG in DI water as shown in Supplementary Fig. S1(a). The reaction rate of SOSG (in arbitrary units) corresponding to each concentration is calculated from the gradient of the linear curve in Supplementary Fig. S1(a). This reaction rate is plotted as a function of SOSG concentration in Supplementary Fig. S1(b). The results show that the reaction rate increases linearly with concentration and above a concentration of 4μM, it is almost constant. According to the laws of chemical kinetics the gradient of the curve in supplementary Fig. S2(b) reflects the order of the reaction. In our case, the regime of zero order kinetics is achieved for the SOSG concentration of 4 μM and above. We have therefore used 4 μM of SOSG and no more than 1 μM of VP in subsequent experiments.

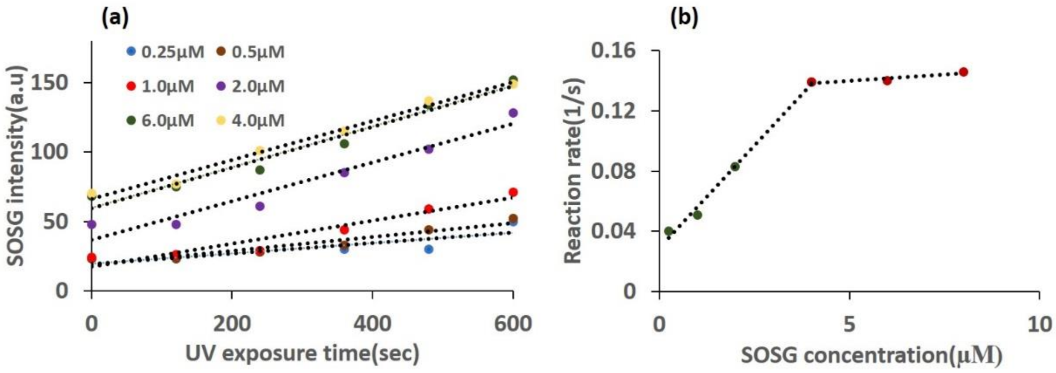

Supplementary Figure S1 *(a) Singlet oxygen generation reaction kinetics for different concentrations of SOSG with 1µM verteporfin; (b) Reaction rate of singlet oxygen generation as a function of SOSG concentration.*

S3. Inner filter correction of SOSG fluorescence

Fluorescence intensity measured by a spectrometer may not be proportional to the sample concentration due to the inner filter effect [2]. As a result of this effect, the observed fluorescence intensity is not directly proportional to the fluorophore concentration, but it depends on optical density of the sample at the excitation and emission wavelengths. In the present study, we corrected the fluorescence intensity according to the following relationship[3].

$$F_{corr} = F_{obs} \times 10^{\frac{D_{exc}+D_{emi}}{2}} \qquad \text{(S3)}$$

In Equation (S3), F_{corr} and F_{obs} represent the corrected and observed fluorescence intensity, respectively, and D_{exc}, D_{emi} are the optical densities at the excitation and emission wavelengths.

S4. Correction of SOSG fluorescence for variable pH

We observed that that the fluorescence of the SOSG probe is highly pH-dependent, which is a common property of many fluorophores. In the investigations which involve nanoparticles,

this property may produce unexpected variations, because pH may significantly vary solely as a function of nanoparticle density used. Here, we show that the addition of various amounts of nanoparticles in DI water, the pH of the solution changes significantly (decreasing with increasing nanoparticle concentration, Supplementary Fig. S2). As a result of this, the SOSG intensity measured in nanoparticle solutions varies accordingly. To account for that, the SOSG intensity in each experiment have been corrected accordingly, to reflect the actual pH conditions produced by the nanoparticles.

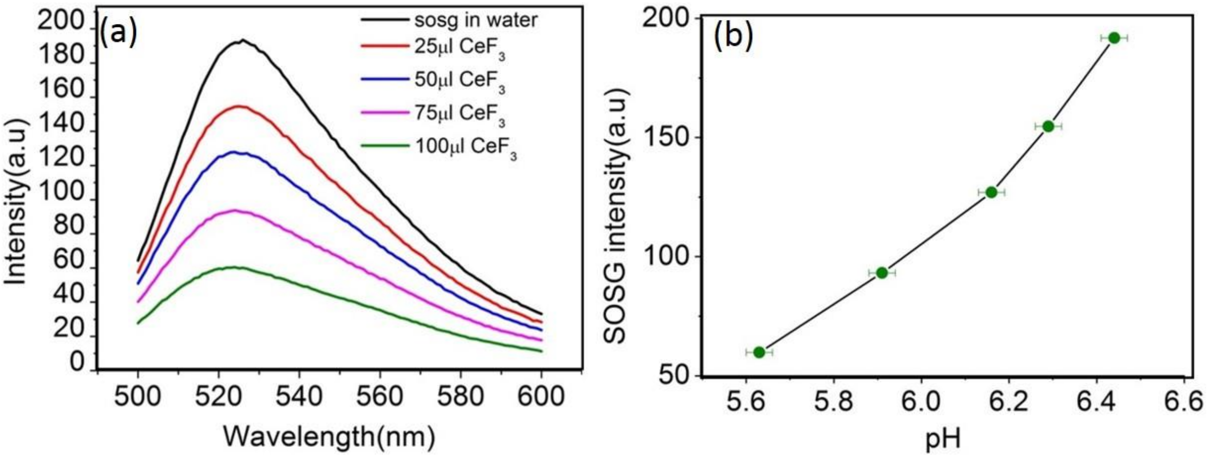

Supplementary Figure S2. *(a) Variations of SOSG emission intensity with 488 nm excitation in DI water as well as with different concentration of nanoparticle in DI water. (b) Change in SOSG intensity with variation in pH caused by variation of* CeF_3 *concentration.*

S5. Determination of the number of UV photons absorbed by the PpIX and calibration of SOSG fluorescence

A mixture of 1 µM of PpIX with 4µM SOSG is held in cuvette and the UV LED irradiating PpIX is mounted in such a way that light is incident from the top of the cuvette. A control sample with 4 µM of pure SOSG has been separately prepared. The fluorescence intensity of SOSG, $I_{SOSG}\ (t_{UV})$ as a function of UV exposure time t_{UV} corrected for the effect of UV light on pure SOSG is plotted as shown in Supplementary Fig. S3(a).

The number of UV photons absorbed in the cuvette as a function of UV exposure time ($N_{UV}(t_{UV})$) is determined as:

$$N_{uv}(t_{UV}) = \frac{P}{E} * F * t_{UV} \tag{S4}$$

where P is the optical power detected on the surface of the sample, E is the energy of 365 nm UV photons and t_{UV} represents the time of UV exposure. F is the absorption factor calculated using the equation

$$F = 1 - e^{-\alpha\rho l} \tag{S5}$$

where α is the molar attenuation coefficient of PpIX, measured separately, ρ is the density of PpIX and l is the path length. Using Equations S4 and S5, we calculated the number of UV photons absorbed by the PpIX for different exposure times, $N_{UV}(t_{UV})$. This quantity is plotted against time as shown in Supplementary Fig. S3 (b).

The SOQY of PpIX in water (the ratio of the number of singlet oxygen molecules generated to the number of visible photons absorbed) has been determined in the literature to be 0.56[4]. Using this value and the number of UV photons absorbed by PpIX, $N_{uv}(t_{UV})$,at varying exposure times, t_{UV}, (Supplementary Fig. S3(b)), we calculate the number of singlet oxygen molecules, $N_s(t_{UV}) = 0.56N_{uv}(t_{UV})$, generated in PpIX at varying UV exposure times. This number of singlet oxygen molecules generated in the PpIX at varying exposure times, $N_s(t_{UV})$, is then plotted against the number of UV photons absorbed during this exposure time, $N_{uv}(t_{UV})$, as shown in Supplementary Fig. S3(c). By bringing together the information on $I_{SOSG}\ (t_{UV})$ from the Supplementary Fig. S3(a) and on $N_S(t_{UV})$, from Supplementary Figure S3(c), we obtain $N_S(I_{SOSG})$, the number of singlet oxygen molecules as a function of the corresponding intensity of SOSG fluorescence; this relationship is shown in Supplementary Fig. S3(d). This provides the calibration of the SOSG signal with respect to

the number of singlet oxygen molecules in our cuvette. The relationship $N_S(I_{SOSG})$ is approximately linear $N_s(I_{SOSG}) = C_F(I_{SOSG} - I_{bckg})$ where the conversion factor C_F is obtained from the slope of Supplementary Fig. S3 (d). The presence of the background term, I_{bckg} is related to the fact that SOSG shows background fluorescence without any singlet oxygen, as indicated elsewhere.

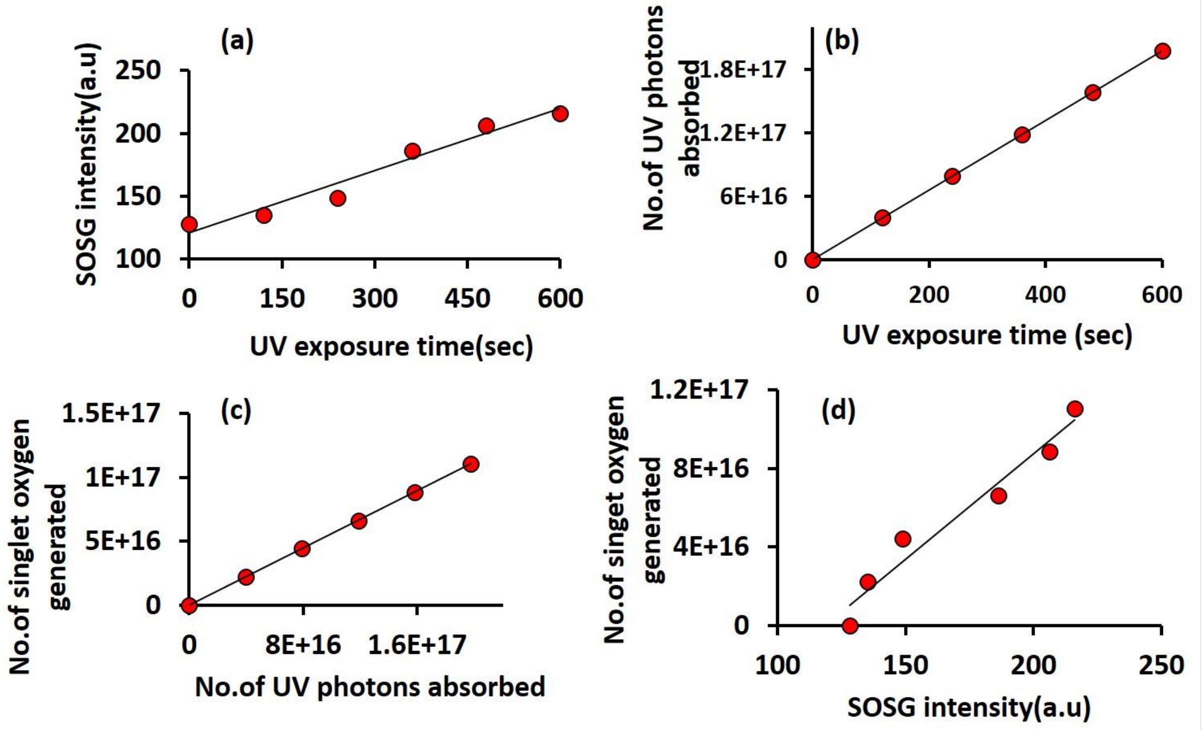

Supplementary Figure S3 *(a) Intensity of SOSG fluorescence at 525 nm from PpIX as a function of exposure time. (b) Number of UV photons absorbed by PpIX as a function of time. (c) Number of singlet oxygen generated versus number of UV photons absorbed. (d) Conversion of fluorescence intensity of SOSG at 525 nm to the number of generated singlet oxygen molecules.*

S6. X-ray Singlet Oxygen Quantum Yield

The X-ray singlet oxygen quantum yield, η, is defined as the number of singlet oxygen molecules generated by the absorption of 1 eV of X-ray photon energy (note a difference

with the definition of SOQY which the number of singlet oxygen molecules per single photon).

To calculate η, we convert the intensity of fluorescence of SOSG for each X-ray exposure time $I_{SOSG}(t_{Xray})$ for Sample C produced by the X-ray exposure (Supplementary Fig. S4(a)) to the corresponding number of singlet oxygen molecules for that exposure time, $N_s(t_{Xray})$, using the approximate expression for $N_s(I_{SOSG})$:

$$N_s(I_{SOSG}) = C_F(I_{SOSG} - I_{bckg})$$

We obtain

$$N_s(t_{xray}) = (I_{SOSG}(t_{xray}) - I_{bckg}) * C_F \quad \text{(S6)}$$

This result, $N_s(t_{xray})$ is plotted as a function of X-ray exposure time, t_{xray} in Supplementary Fig. S4(b).

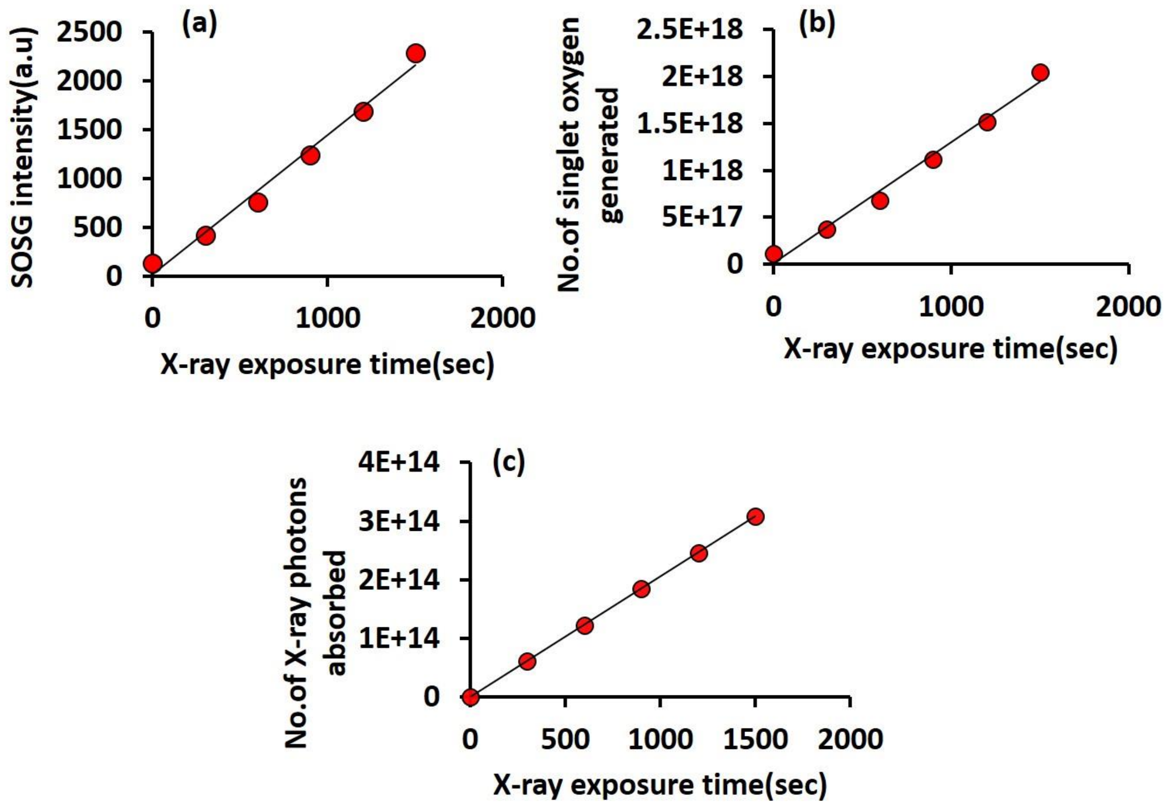

Supplementary Figure S4 *(a). Intensity of SOSG as a function of time for X-ray exposure obtained for Sample C. (b) Number of singlet oxygen molecules generated as a function of time for X-ray exposure. (c) Number of X-ray photons absorbed as a function of X-ray exposure time.*

The number of X-ray photons absorbed, $N_x(t_{xray})$ as a function of time is calculated as

$$N_x(t_{xray}) = \frac{P_w}{E} * F_x * t_{xray} \qquad \text{(S7)}$$

where P_w is the power of the X rays incident on the sample. This power is calculated by considering the electrical parameters of the X-ray tube (tube voltage: 45 kV, tube current: 40 mA) and the efficiency of the X-ray source. By manufacturer specifications the overall efficiency of our X ray source is 0.0088 %. This efficiency is calculated by considering the individual efficiencies of different factors related to the X-ray system as well as the scattering by air. E is the energy of the X-ray photons (for our Cu anode this is 8 keV). F_x is the X-ray absorption factor. The standard equation for this absorption factor is modified as our conjugate consists of water and nanoparticles. The absorption factor in this particular case is given as

$$F_x = \left(1 - e^{-(a+b)l}\right) * \left(\frac{a}{a+b}\right) \qquad \text{(S8)}$$

where a is the product of mass absorption coefficient of CeF_3 nanoparticles and the density of nanoparticles in the sample. We calculated the concentration of nanoparticles in sample C as 320 µM. b is the product of mass absorption coefficient of water and its density. The mass absorption corresponding to each constituent in the nanoparticles as well as water at 8 keV is taken from the NIST database[5]. l is the effective path length of the sample. Using these considerations, the number of X-ray photons absorbed $N_x(t_{xray})$ for each irradiation time is

calculated and plotted as shown in Supplementary Fig. S4(c). The number of singlet oxygen molecules generated by each 8 keV X-ray photon, N_{8keV} is calculated as

$$N_{8keV} = \frac{N_s(t_{xray})}{N_x(t_{xray})} \tag{S9}$$

Using the value obtained from above equation, we can calculate X-ray singlet oxygen quantum yield, η as

$$\eta = N_{8keV} / (8\times10^3) \tag{S10}$$

S7. Dose Partition

The fraction of energy absorbed by the CeF_3 in lung tissue was calculated by considering the dose partition. The amount of light absorbed by CeF_3 and lung tissue are proportional to its density, ρ and is mass absorption coefficient, α;

$$F_{CeF_3} \propto \rho_{CeF_3}\alpha_{CeF_3} \tag{S11}$$

$$F_t \propto \rho_t\alpha_t \tag{S12}$$

Using these relations and the fact that all the light must be either absorbed by the nanoparticle or the tissue, $F_{CeF_3} + F_t$=1, the dose partition F_{CeF_3} that is the fraction absorbed by the nanoparticle in lung tissue can be derived as the following ;

$$F_{CeF_3} = \frac{\rho_{CeF_3}\alpha_{CeF_3}V_p}{\rho_{CeF_3}\alpha_{CeF_3}V_p + \rho_t\alpha_t(1-V_p)} \tag{S13}$$

Here V_p is the nanoparticle volume fraction. The values of mass absorption coefficients were taken from the NIST database [5] .

S8. Cell Viability

We determined the viability of cells exposed to nanoparticle conjugates and gamma radiation. In this study we used normal (HEK 293) and cancer (Panc1) cell lines. The MTS assay was used for testing the viability. Supplementary Fig S5 shows the viability of cells at different radiation doses. Both cell lines shows excellent viability at different radiation dosages.

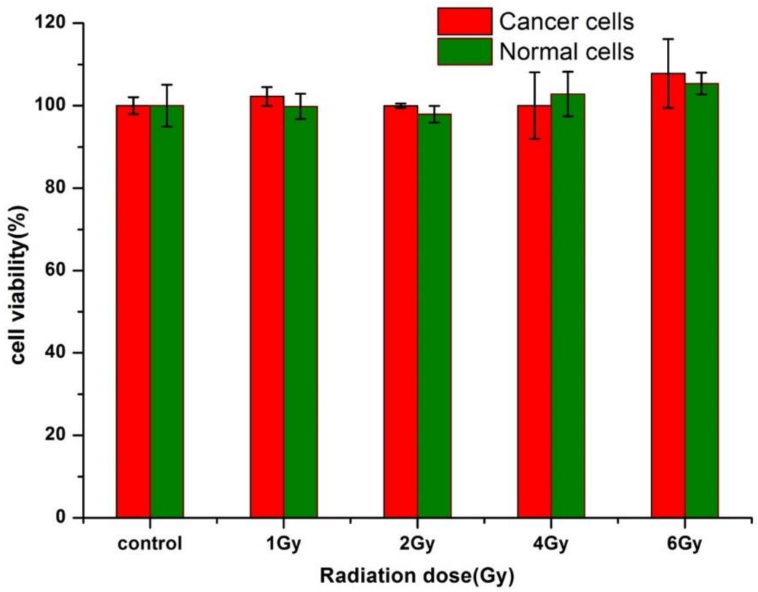

Supplementary Figure S5: *The viability of cancer cells (Panc1) and normal cells(*HEK293) exposed to different radiation doses.

Figure S6 shows the viability of cells treated with CeF_3-VP conjugates. In this experiment, the conjugate C(CeF_3 concentration-320 μM) and its different dilutions were used. The cancer cells showed high viability for all different dilutions of Conjugate C, but normal cells were less han 100% viable with the first two dilutions (1x and 2 x dilution). Hence we chosen the 4x dilution of conjugate C for our PDT experiments.

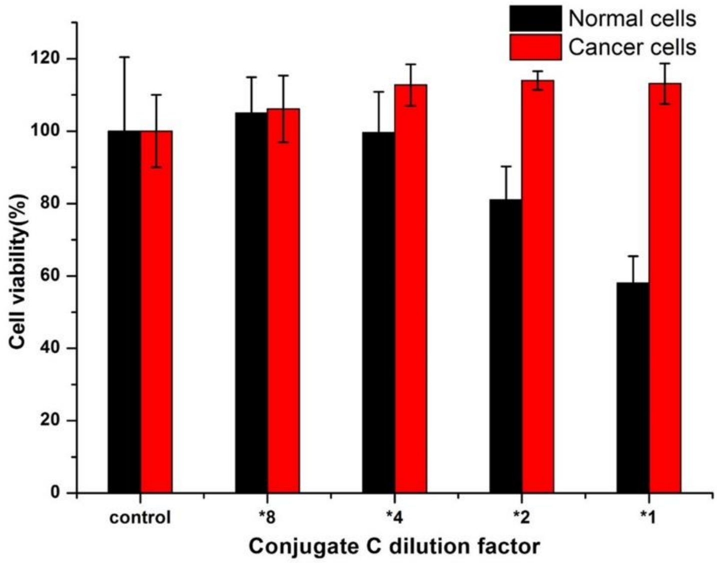

Supplementary Figure S6: *The viability of cancer cells (Panc1) and normal cells (*HEK293) *treated with different dilutions of conjugate C.*

References

1 Lin, H. *et al.* Feasibility study on quantitative measurements of singlet oxygen generation using singlet oxygen sensor green. *J Fluoresc* **23**, 41-47 (2013).

2 Kubista, M., Sjöback, R., Eriksson, S. & Albinsson, B. Experimental correction for the inner-filter effect in fluorescence spectra. *Analyst* **119**, 417-419 (1994).

3 Puchalski, M., Morra, M. & Von Wandruszka, R. Assessment of inner filter effect corrections in fluorimetry. *Fresenius J Anal Chem* **340**, 341-344 (1991).

4 Redmond, R. W. & Gamlin, J. N. A compilation of singlet oxygen yields from biologically relevant molecules. *Photochem and photobiol* **70**, 391-475 (1999).

5 Hubbell, J. H. & Seltzer, S. M. Tables of x-ray mass attenuation coefficients and mass energy-absorption coefficients. *Nat Inst Stand Technol* (1996).
